# Interest of Fluvoxamine as an Add-On to Clozapine in Children With Severe Psychiatric Disorder According to CYP Polymorphisms: Experience From a Case Series

**DOI:** 10.3389/fpsyt.2021.669446

**Published:** 2021-06-21

**Authors:** Camille Berel, Ulysse Mossé, Julien Wils, Lauriane Cousin, Laurent Imbert, Priscille Gerardin, Boris Chaumette, Fabien Lamoureux, Vladimir Ferrafiat

**Affiliations:** ^1^Child and Adolescent Psychiatric Unit, URHEA, CHSR Sotteville les Rouen, Sotteville les Rouen, France; ^2^Department of Pharmacology – Toxicology and Pharmacogenetics, Rouen University Hospital, Rouen, France; ^3^Normandie Univ, UNIROUEN, Inserm U1096, Rouen, France; ^4^Child and Adolescent Psychiatric Department, CHRU Lille, Lille, France; ^5^Child and Adolescent Psychiatric Department, CHU Charles Nicolle, Rouen, France; ^6^Institut de Psychiatrie et Neurosciences de Paris, INSERM UMR 1266, Université de Paris, GDR3557-Institut de Psychiatrie, Paris, France; ^7^GHU Paris Psychiatrie et Neurosciences, Paris, France; ^8^Department of Psychiatry, McGill University, Montreal, QC, Canada

**Keywords:** clozapine, resistant psychiatric disorder, child and adolescent psychiatry, cytochrome polymorphism, fluvoxamine

## Abstract

Despite its drastic efficacy in resistant psychiatric disorders, clozapine remains rarely used in youth due to its side effects. Clozapine plasma level is determined through its metabolism involving several isoforms of cytochromes 450 (CYP450) family. Isoform CYP1A2 appears as a limiting enzyme involved in the metabolism of clozapine, while isoforms 2C19, 2D6, 3A4, and 3A5 also contribute in a minor way. Clozapine efficacy is limited by a significant inter-patient variability in exposure according to CYP's polymorphisms. Clozapine plasma levels may be increased with CYP inhibitors such as fluvoxamine. This drug is a potent enzymatic inhibitor of CYP1A2 and, to a lesser extent, of CYP3A4 and CYP2D6. Hence, in case of CYP's polymorphisms in youth, the use of fluvoxamine as add-on to clozapine could help in reaching clinical and biological efficacy and allowing lower clozapine dosage and a better tolerance profile as it has already been described in adults. We report four pediatric cases with severe psychiatric disorders underlying our experience with CYP polymorphism explorations and the use of fluvoxamine as add-on to clozapine. Our four patients clinically improved after the introduction of fluvoxamine, enhancing clozapine metabolism and therefore the clozapine plasma level within therapeutic range. Despite the interesting results of fluvoxamine, we report a severe issue of tolerance for one patient, emphasizing the need for caution regarding possible drug interactions when fluvoxamine is considered. Hence, we propose a detailed step-by-step multidisciplinary protocol.

## Introduction

In the last years, several studies in children and adolescents with severe psychiatric disorders have demonstrated the effectiveness and safety of clozapine (1). Clozapine is the gold standard for treating treatment-resistant adult patients with schizophrenia after at least two treatment failures (2, 3). For treatment-refractory early-onset schizophrenia (EOS), several studies show that clozapine is the most effective medication both in short-term and maintenance treatment (4–11). Clozapine also showed a better outcome for patients with schizophrenia secondary to 22q11 deletion. Indeed there is a higher prevalence of first-line antipsychotic resistance for those patients (12–14). Data in pediatric population are really sparse and limited to case reports. Clozapine could also be useful for resistant pediatric bipolar disorder (15–17). Several studies have demonstrated the efficacy of clozapine in children and adolescents with aggressive behaviors in psychotic disorders (18, 19) or disruptive disorder (20, 21). Retrospective studies, case series, and case reports suggest the efficacy of clozapine for aggressive behavior in autism spectrum disorder (ASD) or other pervasive developmental disorder (PDD) (22–28). To date, there is no randomized controlled study evaluating this use. Finally, evidence suggesting the efficacy of clozapine in Tourette's syndrome is limited and heterogeneous (29–33).

Despite its high efficacy, *The American Academy of Child and Adolescent Psychiatry* (34) recommends the use of clozapine only after the failure of two or three other antipsychotics in EOS because of its significant side effect profile. Clozapine is an atypical antipsychotic with a complex pharmacological profile: a higher affinity for 5-HT2A receptors than D2 and a lower occupancy D2 compared to other antipsychotic drugs. Whereas, it results in less extrapyramidal effect, tardive dyskinesia, and hyperprolactinemia, it has significant clinical risks such as agranulocytosis, seizures, myocarditis, or cardiometabolic adverse events regardless of the population, whether adult or pediatric (35, 36). Moreover, children and adolescents treated with clozapine or other antipsychotics have significantly higher adverse effects, like weight gain, than the adult population (37). Women and children have higher plasma levels than men and older people (38, 39), and it is likely that these higher levels could increase the rate of adverse events in these populations.

The cytochrome P450 family is a major class of enzyme that mediate biotransformation like oxidation or demethylation of drugs or endogenous substances. This wide family is classified in 18 families and 44 subfamilies (40). Their activities can be affected or influenced by genetic, physiological, pathophysiological, or environmental factors like gender, age, cancer, tobacco, or genetic polymorphism (40, 41). Based on genetic polymorphism variations, there are four cytochrome phenotypical profiles: poor metabolizers, intermediate metabolizers, extensive metabolizers, and ultra-rapid metabolizers. At least 57 different CYP genes have been identified, which can explain a part of the phenotypic contribution (42). At this time, the development of new CYP optical probes, non-optical probes, or fluorogenic probes is being used to better assess interindividual variability (42).

Clozapine plasma level is determined through its metabolism involving hepatic function, several cytochromes (CYP), and the flavin-containing monooxygenase 3. In the liver, clozapine is mainly demethylated to N-desmethylclozapine, which has antipsychotic action (abbreviated NDMC, also called norclozapine), and oxidated to clozapine-N-oxide by several isoforms of the CYP450 family. Isoform CYP1A2 has a major role in the metabolism of NDMC, an active metabolite of the clozapine, while isoforms 2C19, 2D6, 3A4, and 3A5 also contribute in a minor way (43–45). N-desmethylclozapine is an active metabolite acting as a D2 and D3 partial agonist (46) and has affinity for muscarinic, serotonin, and histaminic receptor (47). Clozapine use is limited by a narrow therapeutic range and significant inter-patient variability in exposure according to CYP's polymorphisms. Genetic polymorphisms in the CYP450 genes are thought to account for about 50% of the clozapine plasma level. The remaining 50% mainly depends on the posology, gender, and age (48, 49).

The clozapine plasma levels may also vary according to concomitant treatments, such as CYP inhibitors, including fluvoxamine. Fluvoxamine is a selective serotonin reuptake inhibitor and a sigma-1 receptor agonist (50). This drug is also a potent enzymatic inhibitor of CYP1A2 and, to a lesser extent, of CYP3A4 and CYP2D6. This inhibition activity is effective for a dosage of 50 mg daily in adults (51). The adjunction of fluvoxamine is associated with an increase in clozapine-to-NDMC ratio and a lower NDMC plasma level by inhibiting CYP1A2 (52). The clozapine plasma level is increased by 2–10 with 50 mg/day of fluvoxamine, according to a wide interindividual variability of this effect (53). Associating fluvoxamine to clozapine leads to an improvement of metabolic parameters, with a decrease in weight gain, insulin resistance, and triglyceride levels (54). The clozapine-to-NDMC ratio has been previously shown to be a better indicator of clinical response than the clozapine plasma level in the pediatric population (8). The optimal ratio has not been defined yet; however, a ratio of two seems to be associated with a maximal clinical efficacy (52). Hence, the use of fluvoxamine as add-on to clozapine is thought to help in reaching efficacy with a lower clozapine dosage and a better tolerance profile (52). Consequently, fluvoxamine is widely used in adults to optimize clozapine exposure and to reduce frequent side effects. To the best of our knowledge, to date, only one case report was published about a 16-year-old boy with severe side effects, thus disturbing treatment maintenance. We report our experience with the use of fluvoxamine in four severely ill children who required the introduction of clozapine as a monotherapy at first but which was quickly enhanced by the addition of fluvoxamine.

A protocol pattern specific to our unit (with a slower increase than in adults) was used for the introduction and increase of clozapine dosing. Clozapine was initiated at a daily dose of 12.5 mg at night on day 1 and 25 mg at night on day 2, and then the dosing was increased by steps of 25 mg every 3 days, distributed as follows: lower dosing at morning and higher dosing at night, with a difference of 25–100 mg between the two doses (i.e., BID: 100 mg in the morning and 150 mg at night). The clozapine and N-desmethylclozapine plasma levels, complete blood count (CBC), and electrocardiogram (ECG) were monitored weekly. The targeted clozapine plasma level was 350 ng/mL (55). If the clozapine plasma level was under 350 ng/mL, we systematically searched for all possible causes of low clozapine plasma level such as poor compliance, inflammatory syndrome, tobacco or caffeine consumption, drug (phenytoin, carbamazepine, and rifampicin), and food (char-grilled meat and cruciferous vegetables such as cabbage, Brussels sprouts, turnips, spinach, or broccoli), all known for their possible interaction leading to enzyme induction (55–59). After eliminating these causes, we performed pharmacogenetics testing for CYP. In case of identification of a clinically significant polymorphism known as causal for low clozapine plasma level, we introduced fluvoxamine as add-on to clozapine after a multidisciplinary concertation with the pharmacology department.

All families provided a written informed consent to the use of clozapine for their children after being fully informed of the following: (i) the off-label use and its scientific rationale in the matter of resistant neurodevelopmental disorders, (ii) the clinical requirements and criteria for being considered as having a resistant psychiatric disorder, (iii) the clinical pharmacokinetics of clozapine, (iv) all possible side effects and contraindications known with clozapine, and (v) the specific side effect monitoring scheduled. All families provided a written informed consent for CYP genotyping. In addition, all families provided a written informed consent to the use of fluvoxamine for their children after being fully informed of the following: (i) the off-label use and its scientific rationale in the matter of increasing CYP metabolism in a context of identified polymorphism, (ii) all possible side effects and contraindications known with fluvoxamine, and (iii) the specific side effect monitoring scheduled. No family or legal guardian refused the offer of both treatment or CYP genotyping. This study, E2020-85, was approved by our ethical committee for research on preexisting data at the University Teaching Hospital of Rouen. The committee concluded that this study do not present any ethical issue and any violation of “Loi no. 2012-300 du 5 mars 2012 (dite loi Jarde).” This study is consistent with and conforms to the French law regarding clinical research.

## Case Description

### Case A

A is an 11-year-old Caucasian girl who was born at term after a complicated pregnancy and a threat of preterm birth during the 5th month in a context of physical violence toward her mother. No complication was reported during the delivery. She was eutrophic at birth. The APGAR score was 10/10. She suffers from a permanent moderate hyperphenylalaninemia diagnosed at birth. A diet alone led to acceptable phenylalaninemia levels. This condition severely limits drugs options due to the contraindication of aspartame. Regarding psychomotor development, no delay was found during her 1st year.

She was placed in foster care at the age of 6 years old due to severe neglected behaviors from her parents. She has experienced a very insecure environment leading to an early attachment disorder and anxiety disorder. During primary school, major global learning disabilities were reported. In this context, she was evaluated by a standard metric test (EXALANG 5–8 years). The initial evaluation found severe attentional and behavioral difficulties leading to poor reading and writing skills. Writing rehabilitation was prescribed but stopped due to behavioral disorders. At the age of 9 years old, the Wechsler Intelligence Scale for Children (WISC—IV) was homogeneous with intellectual disability (verbal comprehension index = 69; perceptual reasoning index = 82; processing speed index = 71; full scale IQ = 69). Rapidly, she presented severe outbursts and tantrums leading to hetero-aggressiveness and self-injuries. In the meantime, she started to exhibit many simple motor tics associated with simple verbal tics characterized by facial grimacing, arm jerk, obscene gesture, and scream compatible with Tourette syndrome. The worsening of the Tourette syndrome associated with intellectual deficiency led to many changes in her living environment and many long-term hospitalizations. She was unsuccessfully challenged by risperidone, aripiprazole, fluoxetine, propericiazine, haloperidol, zuclopenthixol, and naltrexone.

**Figure 1:**
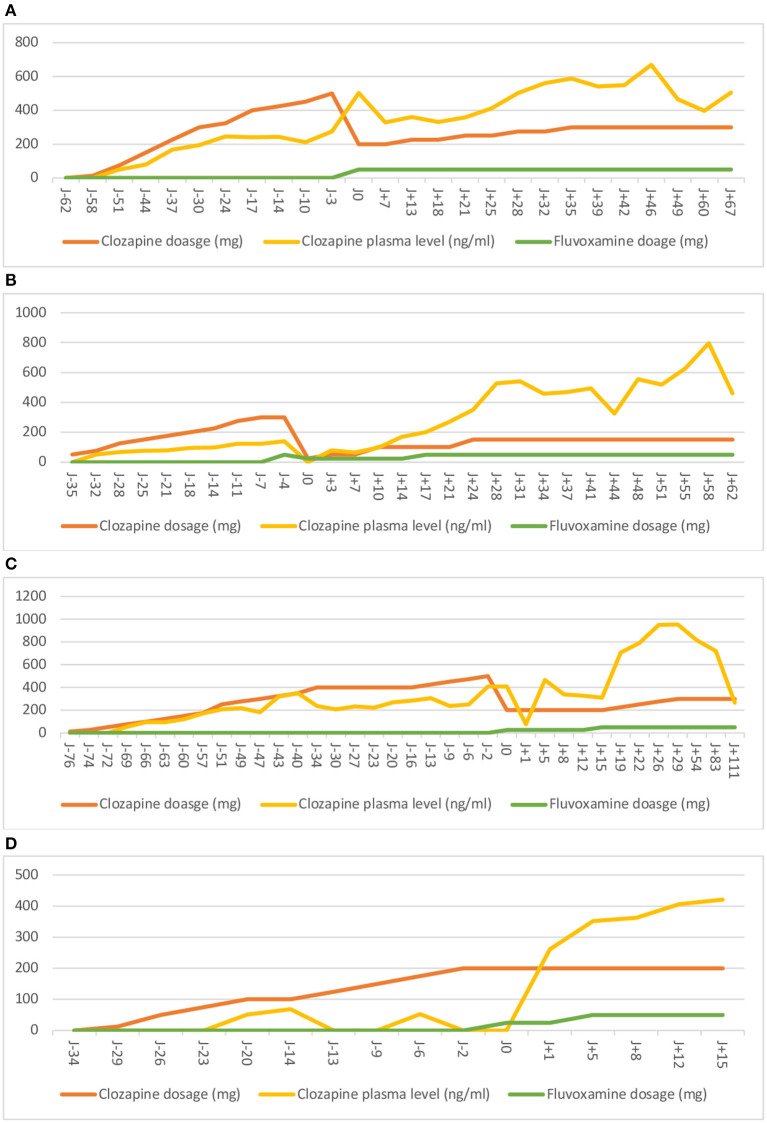
Evolution of clozapine plasma level (ng/mL) for each case. Period of time before and after the introduction of fluvoxamine. J0 represents the day of the introduction of fluvoxamine.

During her last admission in our specialized unit, our clinical assessment underlined the severity of her attachment disorder with severe ambitendency toward her relationship with peers and caregivers, many simple and complex motor/verbal tics, aggressiveness, strong impulsivity, and numerous self-harm behaviors. A clinical examination did not find any abnormality, and the result of a standard blood workup (CBC and liver and kidney function) was normal. Given the overall resistance to atypical and typical antipsychotics, the severity and burden of the automutilations, and the impulsivity, the off-label use of clozapine was considered legitimate by the medical team. We quickly reported low clozapine plasma levels (275 ng/mL), regardless of the progressive increase of clozapine dosage (500 mg/day). After excluding all known food or drug interactions, a pharmacogenetic testing on cytochromes was performed and identified the CYP1A2^*^1F/^*^1F genotype (see Table 1). This genotype is associated with ultra-rapid drug metabolism, therefore explaining the low clozapine plasma levels. We decided to add fluvoxamine to clozapine to inhibit the CYP1A2 subunit and to enhance the clozapine plasma level. Within the next days after fluvoxamine introduction (50 mg/day), we observed a major increase of clozapine plasma level (503 ng/mL) (see Figure 1). Clinically, we reported a drastic improvement regarding automutilations and impulsivity, with a 32% reduction in the ABC—irritability subscale and 33.3% in the ABC—hyperactivity subscale. Regarding tolerance, we did not report any side effect, and no hematological adverse event was reported (see Supplementary Figure 1).

**Table 1:** Pharmacogenetics testing for cytochrome.

	**Case 1**	**Case 2**	**Case 3**	**Case 4**
Genotype CYP1A2	CYP1A2*1F/*1F	CYP1A2*1/*1	CYP1A2*1/*1	CYP1A2*1/*1F
Genotype CYP2D6	CYP2D6*1/*1	CYP2D6*1/*10CYP2D6*1/*41	CYP2D6*1/*1	CYP2D6*1/*4CYP2D6*1/*10
Genotype CYP2C19	CYP2C19*1/*1	CYP2C19*1/*1	CYP2C19*1/*1	CYP2C19*1/*2
Genotype CYP3A5	NA	CYP3A5*1/*1	CYP3A5*3/*3	CYP3A5*3/*3
Genotype CYP3A4	CYP3A4*1/*1	CYP3A4*1/*1	CYP3A4*1/*1	CYP3A4*1/*1
Genotype CYP2C9	NA	NA	CYP2C9*1/*3	CYP2C9*1/*1
Predicted phenotype	CYP1A2: ultra-rapid metabolizer CYP2D6: extensive metabolizer CYP2C19: extensive or rapid metabolizer CYP3A5: NA CYP3A4: extensive or rapid metabolizer CYP2C9: NA	CYP1A2: extensive metabolizer CYP2D6: intermediate metabolizer CYP2C19: extensive or rapid metabolizer CYP3A5: ultra-rapid metabolizer CYP3A4: extensive or rapid metabolizer CYP2C9: NA	CYP1A2: extensive metabolizer CYP2D6: extensive metabolizer CYP2C19: extensive or rapid metabolizer CYP3A5: slow metabolizer CYP3A4: extensive or rapid metabolizer CYP2C9: intermediate to slow metabolizer	CYP1A2: ultra-rapid metabolizer CYP2D6: intermediate to slow metabolizer CYP2C19: intermediate to slow metabolizer CYP3A5: slow metabolizer CYP3A4: extensive or rapid metabolizer CYP2C9: extensive or rapid metabolizer

*NA, not available; CYP, cytochrome*.

### Case B

B is a 9-year-old African boy who was born at term after a normal pregnancy. He was placed in foster care at the age of 3 years old due to severe physical and psychological violence within the family and severe neglected care. He barely had contact with his mother and his father. His family background is marked with a psychotic disorder in one first-degree relative. He quickly presented a delay in language and motor skills. An ear, nose, and throat (ENT) exploration and audiometry were performed at the age of 4 years old without any abnormalities. He did not receive speech therapy or psychomotor therapy. His academic performances were severely impaired due to his global and severe neurodevelopmental delay. School was quickly impossible and interrupted due to repetitive admission in psychiatry for tantrums and severe outbursts. Around the age of 8 years old, B presented an increase of behavioral disorders occurring at school and at home, leading to several hospitalizations for severe outbursts, self-harm, and aggressive behaviors. At the same time, he reported a progressive onset of visual and auditory hallucinations. Risperidone was initiated at 2 mg/day but without any efficacy.

At the admission in our unit, B presented delusional ideas, hallucinations, and mental automatism. He exhibited a disorganized and dissociative motor behavior, disorganized processes of thinking with speech disorder, and cognitive impairment. A clinical examination did not find any abnormality, and the result of a standard blood workup (CBC and liver and kidney function) was normal. After an extensive screening panel (see Supplementary Figure 2 for details of the screening panel), no organic cause was retrieved, and the diagnosis of EOS was retained. Aripiprazole was introduced at 15 mg/day and associated with levomepromazine at 15 mg/day. However, the patient's tolerance was questioned with a severe sedation and a worsening of delusions and hallucinations. The aripiprazole plasma level showed an overdose at 835 ng/mL (therapeutic reference range: 150–500 ng/mL) regardless of the proper dosage–weight ratio. Aripiprazole was then switched to haloperidol gradually at up to 1.5 mg/day. There was no improvement regarding positive symptoms. The haloperidol and levomepromazine plasma levels showed an underdose, respectively, at 1.1 and 5 ng/mL. After excluding all known food or drug interactions, a heterogenous genotype CYP metabolism was suspected. Pharmacogenetic testing for cytochromes was performed, and it identified CYP2D6^*^1/^*^10 and CYP2D6^*^1/^*^41 heterozygous genotypes and a CYP3A5^*^1/^*^1 homozygous genotype (see Table 1). These genotypes are associated with a partial deficiency in CYP2D6 activity and an ultra-rapid metabolizer phenotype CYP3A5, respectively. Therefore, these genotypes are genetic explanations of the high aripiprazole plasma level and low haloperidol plasma level. Given the resistance to two atypical antipsychotics and one typical antipsychotic and the severity and the burden of the psychotic features, the off-label use of clozapine was legitimate. However, during a progressive increase of clozapine dosage (300 mg/day), we reported a low clozapine plasma level (124 ng/mL). After a multidisciplinary staff consensus, we decided to add on fluvoxamine to clozapine. A few days after fluvoxamine's introduction (50 mg/day), the patient presented an extreme sedation and QTc prolongation, requiring a transfer to the pediatric cardiology department for continuous monitoring and discontinuation of the drugs. Indeed a relatively high levomepromazine plasma level (140 ng/mL; therapeutic reference range in adults: 5–25 ng/mL) was retrieved due to CYP2D6 inhibition by fluvoxamine. The clozapine plasma level was low (139 ng/mL). Therefore, the extreme sedation was secondary to an overdose of levomepromazine, leading to the discontinuation of the drug. Clozapine was introduced once again in association with a low dose of fluvoxamine (25 mg/day). Within the next days after fluvoxamine's introduction, the plasma clozapine level dramatically increased (see Figure 1), which was associated with an important improvement of the clinical state regarding positive symptoms (66% reduction in the SAPS) and a reduction of aggressive behavior. Regarding tolerance, besides the drug interactions described above, we did not report any other side effect, and no hematological adverse event was reported (see Supplementary Figure 1).

### Case C

C is a 14-year-old adolescent (Caucasian/Indian ancestry) born at term by cesarean section due to fetal bradycardia. He is the only son of divorced parents. His family history includes a depressive disorder in two first- and second-degree relatives and a psychotic disorder in a second-degree relative. He presented language delay. He subsequently received speech therapy. C exhibited significant learning delays, particularly in terms of graphical skills with dyspraxia, and difficulties in attention, memory, and processing instructions. He also received psychomotor rehabilitation. He went through a standard academic course until secondary school. However, he quickly showed significant absenteeism and dropped out of school due to his psychiatric symptomatology.

His psychiatric follow-up started at the age of 3 years old due to his difficulties in interacting with his peers and social withdrawal. A diagnosis of ASD was made when he was 12 years old. At the age of 13 years old, C was hospitalized at the request of his psychiatrist when negative and positive symptoms appeared. Aripiprazole, at 10 mg/day, was initiated at that time. After discharge and due to a sustained stabilization of the positive symptoms, a diagnosis of ADHD, comorbid to EOS, was possible. Therefore, methylphenidate, at 10 mg/day, was introduced, allowing an improvement of cognitive functions. Unfortunately, C promptly stopped the treatment. A few months later, he presented significant disturbances in the processes of thinking as well as mystical and persecution delusions. After his admission to our unit, C exhibited negative symptoms with apragmatism, social withdrawal, and marked emotional blunting. The positive symptoms were sub-acute and mild, but C was reluctant to express them due to egosyntonia. A clinical examination did not find any abnormality, and the result of a standard blood work up (CBC and liver and kidney function) was normal. After an extensive screening panel (see Supplementary Figure 2 for details of the screening panel), no organic causes were identified, and the diagnosis of EOS was retained with a probable pre-morbid ASD condition. Amisulpride was then initiated. However, at 1 week after the introduction, he presented asymptomatic hyperprolactinemia (80 ng/mL; therapeutic reference range: <15 ng/mL), requiring the discontinuation of amisulpride. His prolactin levels were normalized within 1 month after the intervention. A new introduction of aripiprazole at 15 mg/day allowed a regression of delusions and hallucinations, with a good tolerance. After his discharge from the hospital, C benefited from a neurocognitive rehabilitation program and adolescent daycare clinic. After 6 months, he exhibited a progressive increase of delusions and hallucinations, leading to a switch from aripiprazole to haloperidol at 1.5 mg/day. At that point, C was admitted again in our unit. He reported a poor therapeutic compliance, leading to a worsening of delusions (mystical, filiation, and persecution) and cenesthesic hallucinations with a total adherence. Given that the EOS was resistant to two atypical antipsychotics and one typical antipsychotic and the severity and burden of the psychotic features, the off-label use of clozapine was legitimate. However, we reported fluctuating low clozapine plasma levels (from 248 to 407 ng/mL) with clozapine dosage between 400 and 500 mg/day and a total lack of clinical response. After excluding all known food or drug interactions, a pharmacogenetic testing on cytochromes was performed and identified a CYP2C9^*^1/^*^3 heterozygous genotype (see Table 1). This genotype is associated with a decreased CYP2C9 activity that could partially explain the low clozapine plasma levels. Given the major lack of clinical response and other possible unidentified polymorphisms associated to CYP2C9^*^1/^*^3 heterozygous genotype, we decided to add on fluvoxamine to clozapine. At 5 days after fluvoxamine's introduction (starting at 25 to 50 mg/day), a first increase in clozapine plasma level (467 ng/mL) was observed (see Figure 1). Clinically, we underlined a drastic improvement regarding positive symptoms with a 63.3% reduction in the SAPS. Regarding tolerance, we did not report any side effect, and no hematological adverse event was reported (see Supplementary Figure 1).

### Case D

D is an 11-year-old Caucasian boy who was born at term after a normal pregnancy. He was diagnosed with a mild ASD at the age of 8 years old. The results of genetic testing (fragile X syndrome, karyotyping, and CGH array), metabolic panel, and ENT exploration were negative. His family background is marked by intellectual disability in 1 s-degree relative. He is living with his parents and his brother. Autonomy was partial, and communication skills were characterized by several simple words and short answers to questions.

In January 2019, the parents described a sudden and brutal increase of abnormal movements, including turning on himself, grimacing, and new stereotypies. He also exhibited sudden behavioral changes and became aggressive, withdrawn, and anxious. He started to speak less, presented enuresis, refused to eat, with a loss of weight, and slept poorly. These behavioral changes led to dropping out from school. Several stress factors during this period have occurred in the last months: moved to a new house 4 months before, suspicion of bullied stress at school, and hand–foot–mouth disease 2 months before. Risperidone and fluoxetine were introduced but without any efficacy.

He was admitted in our unit on July 2019 for mixed catatonia with psychomotor agitation, stereotypes, automatic compulsive movements, grimacing, posturing, and negativism. We reported waxy flexibility, catalepsy, and echolalia at clinical examination. The Pediatric Catatonia Rating Scale (PCRS) score was 29. We slowly increased lorazepam to 16.5 mg/day, allowing the catatonic syndrome to stabilize (PCRS score of 10). A clinical examination did not find any abnormality, and the result of a standard blood workup (CBC and liver and kidney function) was normal. An extensive screening panel was performed (see Supplementary Figure 2 for details of the screening panel). No abnormalities were found. However, there was a persistence of aggressive behaviors, screaming, and crying associated with significant anxiety. Aripiprazole, at 2.5 mg/day, was introduced. Prazosin was also introduced because of the suspicion of a post-traumatic disorder and the presence of repetitive nightmares and sleep disturbance. He presented no adverse event. After 3 months of hospitalization, catatonia and general anxiety disorder were stabilized with a PCRS score at 2, allowing his discharge under 13.5 mg/day of lorazepam. However, after returning home, he still refused to go to school or leave the house. He did not want to be apart from his mother and refused to let people go home. He presented a loss of interest, withdrawal, and aggressive behavior. A month later, he was admitted again.

Given the resistance to two atypical antipsychotics and the severity and burden of anxiety and aggressive behavior, the off-label use of clozapine was legitimate. However, during this progressive increase of clozapine dosage (200 mg/day), the clozapine plasma level did not reach the therapeutic range (<50 ng/mL). After excluding all known food or drug interactions, a pharmacogenetic testing on cytochromes was performed and identified a CYP1A2^*^1/^*^1F heterozygous genotype, a CYP2D6^*^1/^*^4 and CYP2D6^*^1/^*^10 heterozygous genotype, and a CYP2C19^*^1/^*^2 heterozygous genotype (see Table 1). These genotypes are associated with an ultra-rapid drug metabolism, therefore explaining the low clozapine plasma level. These polymorphisms can also explain the lack of improvement of the previous treatments. We decided to add fluvoxamine to clozapine. At 1 day after fluvoxamine's introduction (starting at 25–50 mg/day), a drastic increase in clozapine plasma level (261 ng/mL) was observed (see Figure 1). Clinically, we underlined a drastic improvement regarding aggressive behavior, with a 100% reduction in the ABC—irritability subscale and 90% in the ABC—hyperactivity subscale. Regarding tolerance, we did not report any side effect; a repeat blood sample was not a reason to stop the treatment, and no hematological adverse event was reported (see Supplementary Figure 1).

All demographics and clinical characteristics of each patient are summarized in Table 2.

**Table 2:** Demographics, diagnosis, clinical response, and treatments.

	**Case 1**	**Case 2**	**Case 3**	**Case 4**
Sex	Female	Male	Male	Male
Age (years)	11	9	14	10
BMI (kg/m^2^)	15	23	16,6	14,2
Ethnic origin	Caucasian	African	Caucasian/Indian	Caucasian
Diagnosis (DSM-V)	Tourette syndrome PDD	EOS	EOS	ASD
Clinical response	Positive response on irritability hyperactivity and aggressive behavior	Drastic response on hallucinations and delusions	Drastic response on hallucinations and delusions	Positive response on irritability hyperactivity and aggressive behavior
Mean score difference of clinical scales(ABC and SAPS)	ABC scores Δirritability: 32% Δhyperactivity: 33.3%	SAPSΔ: 66%	SAPS Δ: 66.3%	ABC scoresΔirritability: 100%Δhyperactivity: 90%
Prior psychotropic treatments	Risperidone Aripiprazole Fluoxetine Propericiazine Haloperidol Zuclopenthioxol Naltrexone	RisperidoneAripiprazoleHaloperidol	Aripiprazole Amisulpride Methylphenidate Haloperidol	RisperidoneFluoxetineAripiprazole
Treatments maintained during fluvoxamine introduction	Clozapine Pimozide Lorazepam	Clozapine	Clozapine	ClozapinePrazosineNefopamLorazepam

*ABC, Aberrant Behavior Checklist; ASD, autism spectrum disorder; BMI, body mass index; DSM-V, The Diagnostic and Statistical Manual of Mental Disorders—V; EOS, early-onset schizophrenia; PDD, pervasive developmental disorder; SAPS, Scale for the Assessment of Positive Symptoms*.

## Discussion

To our knowledge, this case series is the first detailed report of the pediatric use of fluvoxamine to enhance clozapine. Our four patients clinically improved after the introduction of fluvoxamine as add-on to clozapine, enhancing its metabolism and therefore the plasma level within clozapine concentration's therapeutic range. Tolerance was good, except for case 2. However, this could be attributed to a drug–drug interaction since it did not occur when the combination of fluvoxamine and clozapine was introduced again. It is important to stress that all our patients presented severe and resistant psychiatric disorders (EOS, ASD, and PDD/Tourette syndrome), which justified the initial use of clozapine. It is clear that those patients already represented a therapeutic challenge, as their psychiatric features could not be improved after several therapeutic options. Furthermore, those patients had initially shown no sign of improvement since the introduction of clozapine, with extremely low or negative plasma levels. We would like to discuss three main points: (1) the specifics of the interesting use of clozapine in severe pediatric psychiatric disorders, (2) the importance of exploring CYP polymorphism profiles in severe psychiatric patients, and (3) the encouraging use of fluvoxamine for a customized management of clozapine.

Among the severe psychiatric disorders supporting clozapine, early-onset schizophrenia is a rare form of schizophrenia defined by an onset before the age of 18 years old, with a prevalence of <1/10,000 children in the general population (60).Two systematic reviews of the literature (6, 7) have shown the superior efficacy of clozapine in resistant EOS. Clozapine improves all EOS features, including negative symptoms (8, 10), allowing a reduction in the number and period of hospitalizations (6). In our study, clozapine, combined with fluvoxamine, reduced the SAPS score in case reports 2 and 3, respectively, by 66 and 66.3%, showing a therapeutic response. The aggressive behavior is a significant concern, with a major burden on the quality of life for patients with ASD and their caregivers. A retrospective review of 135 individuals with ASD demonstrated that 39.5% (*n* = 53) of the individuals met the criteria for drug-refractory behaviors (defined by trials of risperidone and aripiprazole or three or more psychotropic drugs targeting irritability) (61). Clozapine has received very little attention despite open-label studies suggesting its potential efficacy on aggressive behavior in ASD (22–25, 62) and intellectual disability (28, 63). In our study, clozapine improved the ABC—irritability sub-scores by 32% in case report 1 (severe neurodevelopmental disorder) and 100% in case report 4 (ASD). Despite its drastic efficacy, clozapine remains rarely used in youth due to its side effects, particularly hematologic toxicity with the risk of agranulocytosis. The risk associated with clozapine can be minimized and better apprehended with a careful and close monitoring in severely ill youth who present psychotic symptoms resistant to conventional treatments (64). Among the four studied patients, clozapine was well-tolerated, except in case report 2, according to a pre-existing drug–drug interaction involving levomepromazine that was corrected afterwards. None of the four patients had agranulocytosis, myocarditis, or seizures even after the add-on of fluvoxamine (see Supplementary Figure 1).

Several CYP450 enzymes are involved in clozapine metabolism. Clozapine pharmacokinetics may vary according to several functional single-nucleotide polymorphisms (SNPs). There is a growing interest in the area of pharmacogenomics, but the prevalence of these SNPs is usually studied in adults. To date and to the best of our knowledge, data dealing with the pharmacogenetics of antipsychotics in the pediatric population are lacking. The hepatic metabolism of clozapine is complex and involves several enzymes (55, 65, 66). Of these, the CYP1A2 isoform plays a major role, and the allele CYP1A2^*^1F (rs762551) is associated with an ultra-rapid metabolism of substrates, including clozapine and olanzapine (67). Ultra-rapid metabolism may increase drug clearance and lead to low plasma levels of various treatments such as clozapine, inciting clinicians to conclude falsely about treatment inefficiency. Some authors found a prevalence of around 51.3% for this genotype (68). Clozapine is also metabolized in the same manner by CYP3A4 and CYP2C19. CYP3A4 polymorphism exhibits considerable interethnic variability, and CYP3A4^*^22 (rs35599367) appears as the main deleterious SNP in Caucasians (54). The CYP2C19 isoform is also highly polymorphic, and CYP2C19^*^2 (rs4244285) leads to a reduced metabolic activity, while CYP2C19^*^17 (rs12248560) is the main variant associated with an increased activity of this enzyme. To a lesser extent, the CYP2D6 and CYP2C9 isoforms have been involved in the metabolism of clozapine. An increased activity of CYP2D6 is described in 0.7–5.6% of Caucasians (CYP2D6 copy number variation, *i.e*., CYP2D6^*^xN), while about 30% are carrying deleterious allele(s) associated to a reduced CYP2D6 activity (69). These deleterious variants mainly include CYP2D6^*^3 (rs35742686), CYP2D6^*^4 (rs3892097), CYP2D6^*^5 (CNV, gene deletion), CYP2D6^*^6 (rs5030655), CYP2D6^*^9 (rs5030656), CYP2D6^*^10 (rs1065852), and CYP2D6^*^41 (rs28371725). Regarding the CYP2C9 isoform, CYP2C9^*^2 (rs1799853) and CYP2C9^*^3 (rs1057910) are the main deleterious variants found in Caucasians. Moreover, clozapine and its metabolites have been described as substrates of the highly polymorphic P-glycoprotein (P-gp) (*MDR1*) drug efflux transporters. However, the effects of the P-gp variants on clozapine pharmacokinetics and pharmacodynamics remain unclear (65). In the present case series, two patients exhibited CYP1A2 ultra-rapid metabolizer genotype for clozapine. Another case presented an atypical metabolism profile based on the extremely low clozapine plasma level, while no mutations were found. Finally, case number 3 illustrates a limit of such targeted pharmacogenetic approach as no well-characterized polymorphism could explain the low clozapine plasma levels. In this specific situation, the decision of adding fluvoxamine was supported by the following conditions: (i) the absence of all other possible etiologies (toxic, diet, and drugs) which could have enhanced CYP450, (ii) the persistence of no clinical response to clozapine, (iii) the persistence of an undetectable clozapine plasma level, (iv) the crucial need to improve the psychotic symptoms, and (v) the fact that the absence of well-known mutations does not exclude other possible mutations not yet identified. This case raised two questions: firstly, for which clinical situation should we proceed with CYP genotyping? Secondly, when should we consider the addition of fluvoxamine to clozapine therapy?

**Figure 2:**
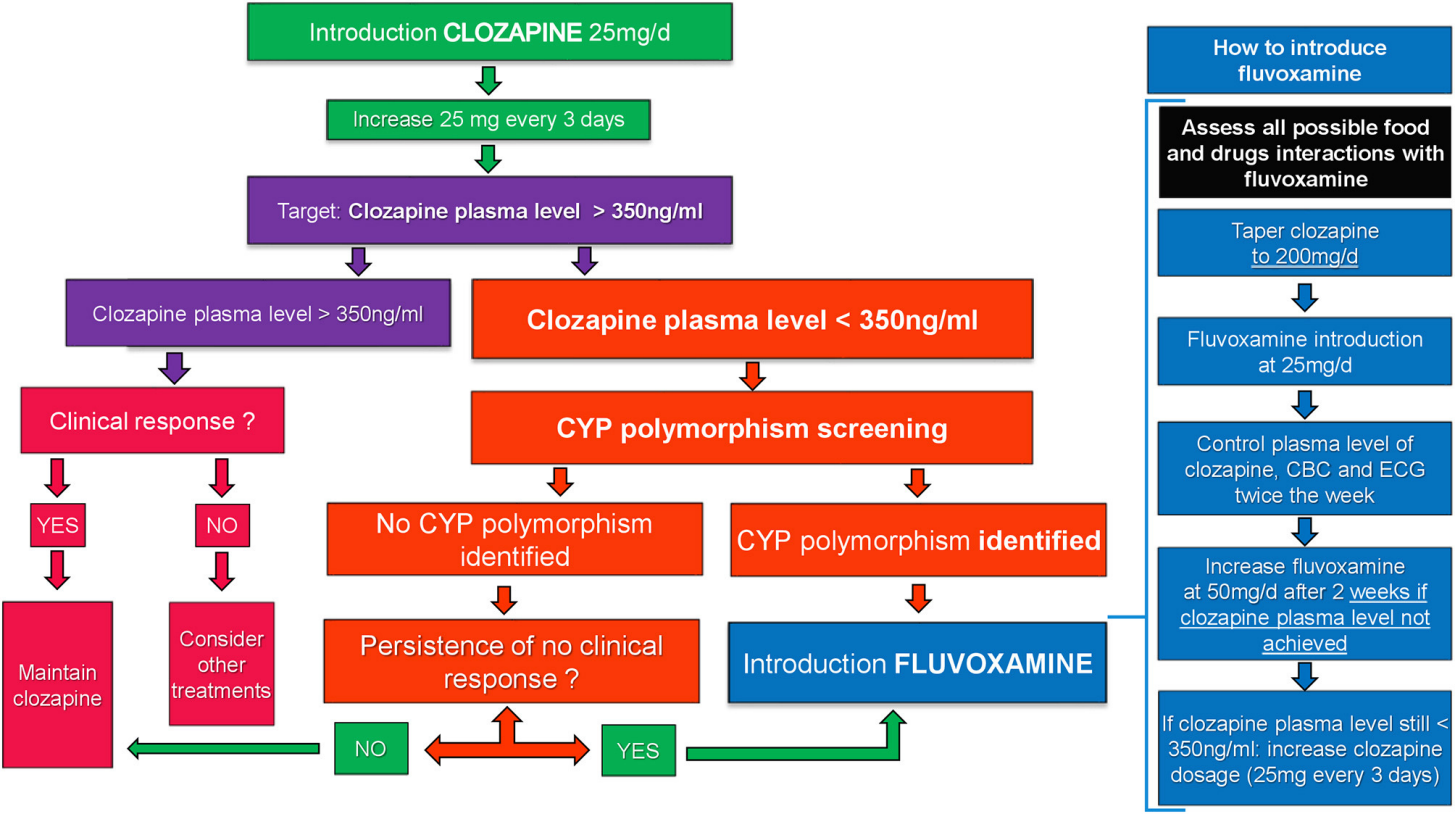
Algorithm.

The clozapine plasma level should be monitored weekly and reach a threshold of 350 ng/mL in order to expect efficacy (55). However, as discussed above, CYP450 polymorphisms can result in a low plasma level and the ineffectiveness of clozapine. Genotyping should be considered when persistent low (under 350 ng/mL) or negative clozapine plasma levels occur in regards of proper dosage (300–600 mg/day) after having excluded all of the other causes listed above and when patients do not exhibit any clinical response and improvement. If both situations are present, fluvoxamine adjunction should be seriously considered (see Figure 2). The adding on of fluvoxamine to clozapine increases the clozapine plasma level and the clozapine/norclozapine ratio by inhibiting cytochromes (52). This leads to an increase in therapeutic effect and reduces adverse effects with a lower dosage (52, 54). Our four patients showed a drastic increase of clozapine plasma level and ratio in clozapine/desmethylclozapine plasma level quickly after the introduction of fluvoxamine at 25 mg (see Figure 1).

Based on our clinical experience and the tolerance issue we faced in case 2, starting fluvoxamine at 50 mg/day following the same pattern with that in the adult population appeared unsuitable and unsafe for the pediatric population, with the risk of a high increase of the clozapine plasma level. Therefore, we reviewed our practice after concertation with the pharmacology department in our institution and proposed a strict protocol for the introduction of fluvoxamine as add-on to clozapine in pediatric patients: (1) drug interactions with fluvoxamine must be carefully assessed in order to identify all possible risks of overdosage of any on-going comedication other than clozapine, (2) the clozapine regimen was adjusted at 200 mg/day in two separate takes (100 mg in the morning and 100 mg in the evening), regardless of the previous dosage, (3) systematically start fluvoxamine at 25 mg/day (half of the dosage used for adults) in order to better control the increase of clozapine plasma levels, (4) the therapeutic drug monitoring of clozapine plasma levels was systematic, with CBC and ECG monitoring twice a week, and (5) still targeting clozapine plasma through levels at 350 ng/mL. If this target is not achieved, we recommended to increase the fluvoxamine regimen at 50 mg/day. If the clozapine plasma level remained systematically under 350 ng/mL after 2 weeks, we proposed to increase clozapine dosage with the same pattern as described above. This protocol is summarized with an algorithm in Figure 2. Once fluvoxamine has been introduced at an effective dose, careful caution must be applied to any new treatment introduced, particularly with enzyme inducers or inhibitors, due to the high risk of drug interactions.

In the absence of an easy access to pharmacogenetic testing, the first possibility is to seek for any national or even international labs that agree to process those tests. Indeed numerous labs offer this expertise at a worldwide scale. Blood sample and DNA are stable enough to be sent through postal service toward the labs. In the case where this option cannot be considered, we suggest the presumptive use of fluvoxamine if the following conditions are met: (i) the existence of severe psychiatric disorder altering the patient's functioning in a severe and lasting manner, (ii) the absence of clinical improvement despite several lines of first- and second-generation antipsychotics, (iii) the persistence of clozapine plasma levels lower than 350 ng/mL despite the increase of clozapine dosages and clozapine-to-NDMC ratio lower than 2, (iv) verifying the absence of any other drugs or substances that could interfere with the metabolism of clozapine, explaining the low clozapine plasma levels, and (v) verifying the absence of any possible interaction between concomitant molecules and fluvoxamine. Only after all these conditions are met and in the absence of other possible alternatives could the introduction of fluvoxamine without genotyping be considered.

However, the presented results should be interpreted in the context of several limitations. First, the number of cases was low and without a long-term follow up after discharge. Second, the absence of double-blind use of fluvoxamine does not allow any validation of its safety in youth. The strengths of this case series must be underlined. This is the first report of fluvoxamine's use in the pediatric population. To our knowledge, no other study took interest on the key role of CYP450 polymorphisms and fluvoxamine in order to better target treatment options for severe psychiatric disorders in youth. Despite the interesting results of fluvoxamine, we report a severe issue of tolerance for one patient (case report number 2), emphasizing the need for caution regarding possible drug interactions when fluvoxamine is considered. Hence, we propose a detailed protocol (see Figure 2). Furthermore, we believe that exploring CYP450 polymorphisms associated to a close therapeutic monitoring is an interesting clinical tool to better understand the low response to drug or clinical resistance and to propose available therapeutic solutions such as fluvoxamine besides the standard monitoring of treatment plasma levels. In the end, a close plasma level monitoring and adjunction of fluvoxamine could lead to a better understanding of clozapine efficacy, a targeted treatment approach, a shorter hospitalization, and less relapse in the future.

## Conclusion

This case series underlines the benefits of clozapine in severe neurodevelopmental disorders. To our knowledge, this is the first study to report the importance of targeting CYP450 polymorphisms to explore the association of clozapine pharmacokinetics and pharmacogenomics and to guide toward a fluvoxamine add-on as a booster in the pediatric population. The results pointed out the positive clinical effects of fluvoxamine as add-on to clozapine in youth with severe neurodevelopmental disorders. From our clinical experience, the use of fluvoxamine add-on to clozapine in youths, as it has already been described in adults, appears useful and interesting but stresses the need for caution regarding drug interactions and for future studies to validate its efficacy and safety on a larger sample.

## Data Availability Statement

All relevant data are included in the article and/or its Supplementary Information files. All other data supporting the study are available from the corresponding author upon request.

## Ethics Statement

Written informed consent was obtained from the individual(s), and minor(s)' legal guardian/next of kin, for the publication of any potentially identifiable images or data included in this article.

## Author Contributions

CB, UM, and VF collected and processed the data and wrote the manuscript. JW, LC, LI, PG, BC, FL, and VF reviewed the literature. CB and UM co-wrote the manuscript. All the authors reviewed the manuscript.

## Conflict of Interest

BC has received speaking fees from Janssen-Cilag. The remaining authors declare that the research was conducted in the absence of any commercial or financial relationships that could be construed as a potential conflict of interest.
