# Supplementary material for: Interest of Fluvoxamine as an Add-On to Clozapine in Children With Severe Psychiatric Disorder According to CYP Polymorphisms: Experience From a Case Series
Source: Front Psychiatry. 2021 Jun 21;12:669446. doi: 10.3389/fpsyt.2021.669446 (PMC8255476; doi:10.3389/fpsyt.2021.669446)
Supplement: Supplementary file 1 [file Data_Sheet_1.PDF]

Supplementary Material

Supplementary Figure 2: Extensive screening panel

	Case 1	Case 2	Case 3	Case 4
**Standard panel**	CBC<br>Ionogram<br>Urea / Creatinine<br>Liver function<br>Lipid function<br>Prolactin<br>Vitamine D<br>Creatinin Kinase	CBC<br>Ionogram<br>Urea / Creatinine<br>Liver function<br>Lipid function<br>Prolactin<br>Vitamin D<br>Creatinine Kinase	CBC<br>Ionogram<br>Urea / Creatinine<br>Liver function<br>Lipid function<br>Prolactin<br>Vitamin D<br>Creatinine Kinase	CBC<br>Ionogram<br>Urea / Creatinine<br>Liver function<br>Lipid function<br>Prolactin<br>Vitamin D<br>Creatinine Kinase<br>Calcium level<br>Albumin
**Metabolic panel**	Ammonia<br>Homocysteine<br>Urine copper<br>Lactate / Pyruvate<br>Plasma and urine<br>Chromatography of amino acids<br>Chromatography of organic acids<br>Oxysterol<br>Cholestanol<br>Phenylalanine	Ammonia<br>Homocysteine<br>Ceruloplasmin<br>Serum copper<br>Urine copper<br>Plasma and urine<br>Chromatography of amino acids<br>Chromatography of organic acids<br>Oxysterol<br>Cholestanol	Ceruloplasmin<br>Serum copper<br>Urine copper<br>Plasma and urine<br>Chromatography of amino acids<br>Chromatography of organic acids<br>Oxysterol<br>Cholestanol	Ammonia<br>Homocysteine<br>Lactate / Pyruvate<br>Ceruloplasmin<br>Serum copper<br>Urine copper<br>Plasma and urine Chromatography of amino acids<br>Chromatography of organic acids<br>Oxysterol<br>Cholestanol<br>Hexosaminidase
**Autoimmune panel**	Antinuclear factor<br>Anti-DNA antibody	Antinuclear factor<br>Anti-DNA antibody	Antinuclear factor<br>Anti-TPO antibody<br>Anti-TG antibody<br>ANCA	Antinuclear factor<br>Anti-DNA antibody<br>Anti-histone antibody<br>Anti-TPO antibody<br>Anti-TG antibody

**Hormonal panel**	TSH	TSH / T4<br>LH/ FSH<br>IGF1<br>Testosterone<br>17-OHP / D4A	TSH<br>T4	TSH
**Lumbar puncture**	NA	NA	NA	Cerebrospinal fluid protein<br>Glycorrhachia<br>Pleocytosis<br>Oligoclonal bands<br>Anti-TG and TPO antibody<br>Anti-NMDAR antibody<br>Anti-GABA antibody<br>Anti-VGKC antibody<br>Anti-GAD antibody<br>Anti-aquaporin antibody<br>Amino acid
**EEG**	Negative	Negative	Negative	Negative
**MRI**	Negative	Negative	Negative	Negative
**Others**		Audiometry test<br>Genetic consultation		Esophagogastroduodenoscopy<br>Dental checkup
