# Supplementary material for: Interest of Fluvoxamine as an Add-On to Clozapine in Children With Severe Psychiatric Disorder According to CYP Polymorphisms: Experience From a Case Series
Source: Front Psychiatry. 2021 Jun 21;12:669446. doi: 10.3389/fpsyt.2021.669446 (PMC8255476; doi:10.3389/fpsyt.2021.669446)

Supplementary Material

Supplementary Figure 1: Evolution of leukocyte count (G/L) before and after introduction of fluvoxamine

(A)

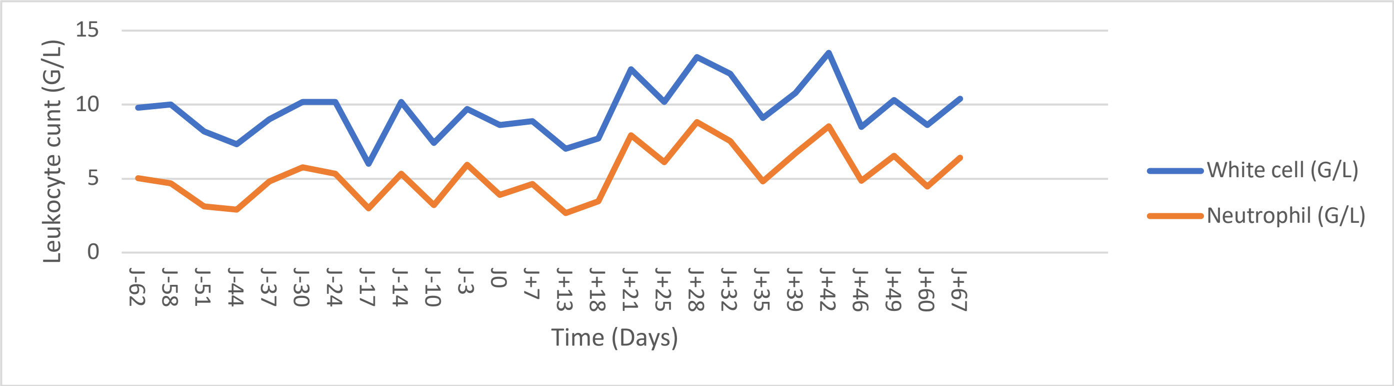

(B)

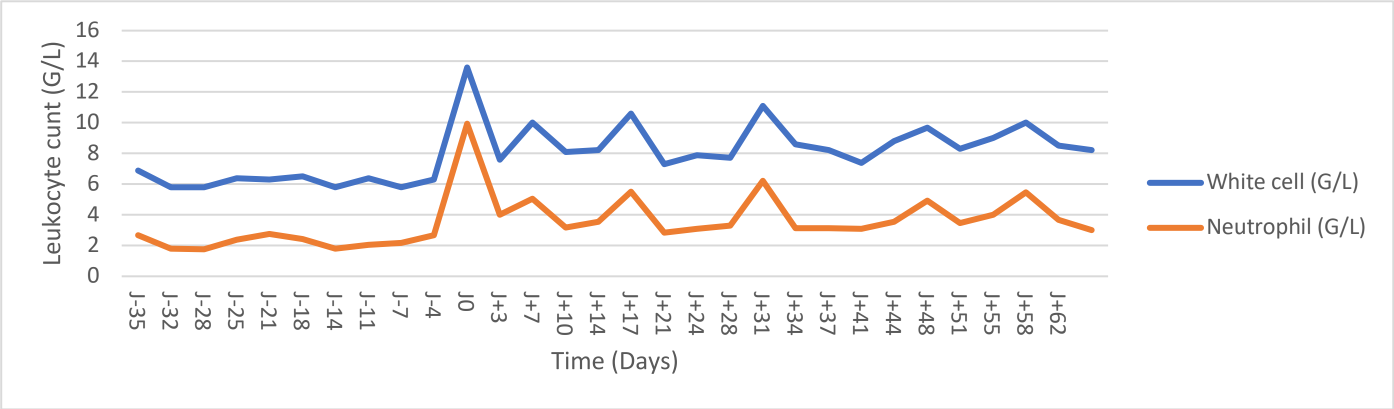

(C)

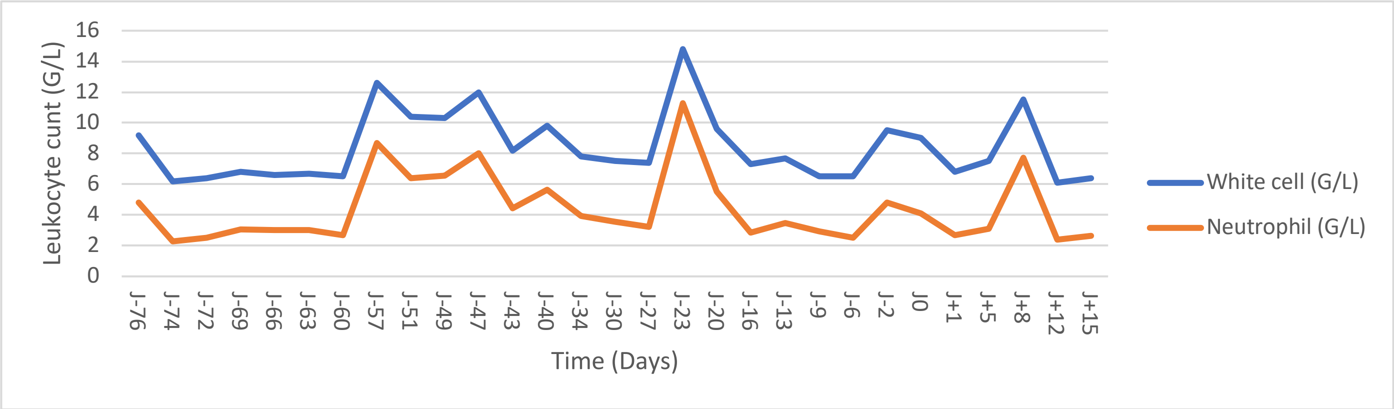

(D)

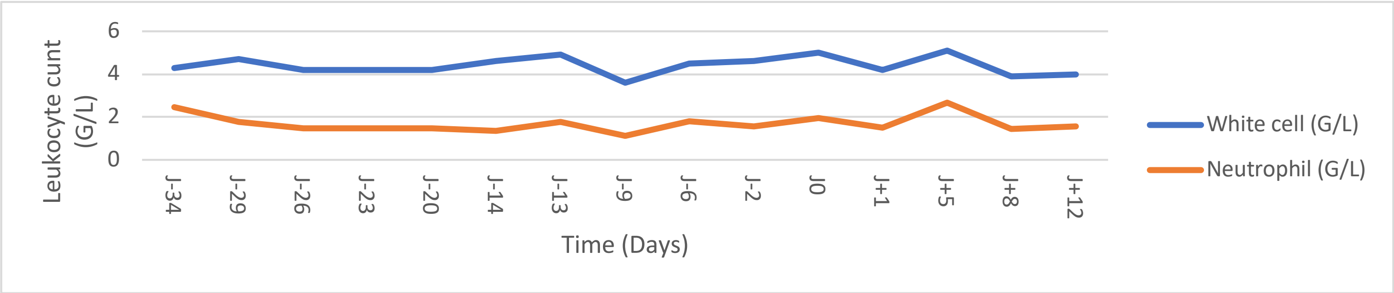

Supplement: Supplementary file 2 [file Data_Sheet_2.PDF]
